# Supplementary material for: Economic and Clinical Benefits of Bivalent Respiratory Syncytial Virus Prefusion F (RSVpreF) Maternal Vaccine for Prevention of RSV in Infants: A Cost-Effectiveness Analysis for Mexico
Source: Vaccines (Basel). 2025 Jan 16;13(1):77. doi: 10.3390/vaccines13010077 (PMC11769006; doi:10.3390/vaccines13010077)
Supplement: Supplementary file 1 [file vaccines-13-00077-s001.zip › vaccines-3368342-supplementary.pdf]

Supplementary

Table S1. Relative risk of RSV encounters, by term status and age in months [17].

Term status	Relative risk of RSV encounters		
	0-< 3 months	3-<6 months	6 to <12 months
Full term (≥ 37 wGA)	1.0	1.0	1.0
Late preterm (32-36 wGA)	1.7	2.5	1.7
Early preterm (28-31 wGA)	0.5	2.4	6.8
Extreme preterm (≤ 27 wGA)	0.5	2.4	6.8

RSV respiratory syncytial virus, *wGA* weeks of gestational age.

Table S2. Distribution of RSV encounters, by calendar month [15].

Calendar month	Distribution of RSV encounters		
	Hospitalization	Emergency department	Outpatient
October	2.5%	2.5%	2.5%
November	19.3%	19.3%	19.3%
December	41.2%	41.2%	41.2%
January	14.7%	14.7%	14.7%
February	11.8%	11.8%	11.8%
March	4.6%	4.6%	4.6%
April	0.8%	0.8%	0.8%
May	0.0%	0.0%	0.0%
June	0.0%	0.0%	0.0%
July	0.0%	0.0%	0.0%
August	0.4%	0.4%	0.4%
September	4.6%	4.6%	4.6%

RSV respiratory syncytial virus.

Table S3. Infant mortality rate (per 1,000 live infants), by age [18].

Infant mortality rate (per 1,000 live infants), by age	Value
< 1 month	6.8
1 - < 2 months	1.0
2 - < 3 months	0.6
3 - < 4 months	0.4
4 - < 5 months	0.3
5 - < 6 months	0.3
6 - < 7 months	0.2
7 - < 8 months	0.2
8 - < 9 months	0.2
9 - < 10 months	0.1
10 - < 11 months	0.1
11 - < 12 months	0.1

Table S4. Relative risk of infant mortality, by term status and age in months [19].

Term status	Relative risk of RSV encounters		
	<1 month	1 to <6 months	6 to <12 months
Full term (≥ 37 wGA)	1.0	1.0	1.0
Late preterm (32-36 wGA)	7.7	3.4	2.6
Early preterm (28-31 wGA)	42.7	9.8	7.3
Extreme preterm (≤ 27 wGA)	482.3	40.6	29.9

RSV respiratory syncytial virus, *wGA* weeks of gestational age.

Table S5. Relative risk of death due to RSVH, by term status [3,20,21].

Term status	Relative risk of death due to RSVH
Full term (≥ 37 wGA)	1
Late preterm (32-36 wGA)	8.0
Early preterm (28-31 wGA)	8.0
Extreme preterm (≤ 27 wGA)	8.0

RSVH RSV hospitalization, *wGA* weeks of gestational age.

Table S6. Distribution of vaccinations, by fetal wGA at time of administration [24].

Fetal wGA	Distribution of vaccinations at time of administration
24	16%
25	12%
26	9%
27	10%
28	9%
29	10%
30	5%
31	7%
32	8%
33	5%
34	2%
35	1%
36	6%

wGA weeks of gestational age.

Table S7. General population health state utility, by age (years) [25].

Age (years)	General population health state utility
1 – 4	0.94
5 – 17	0.94
18 – 49	0.93
50 – 64	0.92
65 – 74	0.91
75 – 84	0.91
85 – 99	0.91

Table S8. Disutility due to RSV and caregiver QALY loss due to RSV [26,27].

Term status	Disutility due to RSV*
Full term (≥ 37 wGA)	0.0157
Late preterm (32-36 wGA)	0.0157
Early preterm (28-31 wGA)	0.0157
Extreme preterm (≤ 27 wGA)	0.0157
**Caregiver QALY loss due to RSV**	0.0066

QALY quality-adjusted life years, *RSV* respiratory syncytial virus, *wGA* weeks of gestational age.
* Disutility was assumed the same irrespective of the setting.
